# Rethinking Immunotherapy Resistance in Melanoma: A Narrative Review of Clinical Patterns, Resistance Mechanisms, and Future Strategies

**DOI:** 10.3390/cancers18172829

**Published:** 2026-09-01

**Authors:** Ali Ghais, Joe Rizkallah, Bahaa El Deen Wehbeh, Youssef Diab, Ali Awada, Mostafa Atwi, Rama Ayoub, Carine Mina, Firas Rammal, Aya Badr, Sary Faraj, Nicole Charbel, Firas Kreidieh

**Affiliations:** 1 Department of Internal Medicine, Virginia Commonwealth University, Richmond, VA 23298, USA; ali.ghais@vcuhealth.org; 2 Division of Hematology and Oncology, Department of Internal Medicine, American University of Beirut, Beirut P.O. Box 11-0236, Lebanon; jr56@aub.edu.lb (J.R.); bhw02@mail.aub.edu (B.E.D.W.); ydd01@mail.aub.edu (Y.D.); ama221@mail.aub.edu (A.A.); ma776@aub.edu.lb (M.A.); ra526@aub.edu.lb (R.A.); cm81@aub.edu.lb (C.M.); fmr08@mail.aub.edu (F.R.); amb75@mail.aub.edu (A.B.); sf67@aub.edu.lb (S.F.); nc47@aub.edu.lb (N.C.)

**Keywords:** melanoma, immune checkpoint inhibitors, immunotherapy resistance, primary resistance, acquired resistance, tumor microenvironment

## Abstract

**Background/Objectives**: Immune checkpoint inhibitors have revolutionized the treatment of advanced melanoma, yet primary and acquired resistance remain major barriers to durable disease control. This narrative review examines the clinical patterns, biological mechanisms, biomarker limitations, and therapeutic implications of immunotherapy failure. **Methods**: A structured search of PubMed, Web of Science, and ClinicalTrials.gov identified relevant studies published from January 2010 to May 2026, supplemented by landmark earlier reports. **Results**: Immunotherapy resistance encompasses heterogeneous patterns, including early progression, oligoprogression, dissociated response, brain-dominant progression, relapse after response, and progression after treatment discontinuation. These clinical phenotypes arise from overlapping mechanisms such as loss of antigen presentation, impaired interferon signaling, oncogenic immune exclusion, melanoma dedifferentiation, suppressive myeloid and regulatory T-cell programs, stromal and vascular barriers, metabolic restriction, and deterioration of tumor-reactive T-cell clones. Conventional response criteria and static biomarkers, including PD-L1 expression and tumor mutational burden, do not adequately capture this complexity because resistance varies across lesions and evolves under treatment pressure. Negative trials of IDO1 inhibition, cytokine intensification, vaccines, and intratumoral immune stimulation further show that biological plausibility and early activity do not guarantee clinical benefit when target engagement, patient enrichment, and endpoint selection are inadequate. **Conclusions**: Immunotherapy resistance in melanoma should be viewed as a variety of biologically distinct clinical states rather than a single endpoint. Future progress will require resistance-matched treatment, longitudinal tissue and circulating tumor DNA assessment, adaptive trial designs, and endpoints aligned with treatment kinetics. This framework may improve salvage selection and interpretation of post-PD-1 studies in clinical practice.

## Simple Summary

Immune checkpoint inhibitors have changed the treatment of advanced melanoma, but not all patients benefit from them, and some tumors progress after an initial response. These failures do not occur in the same way. Some patients progress early, while others develop mixed responses, isolated progression, brain involvement, or relapse after treatment has been stopped. The biological reasons also differ and may include loss of tumor recognition, impaired immune signaling, a suppressive tumor microenvironment, or failure of tumor-reactive T cells. This review brings these clinical patterns and resistance mechanisms together and examines what can be learned from previous unsuccessful trials. Understanding why treatment fails in each setting may help guide more appropriate treatment choices and support the design of future trials that are better matched to the underlying mechanism of resistance.

## 1. Introduction

Immune checkpoint inhibitors (ICIs) have revolutionized the treatment of advanced melanoma [1,2,3]. Although a significant subset of patients achieves durable disease control, this benefit remains unevenly distributed, and resistance to ICIs has become a central challenge in melanoma management [1,4]. Resistance is conventionally divided into primary resistance, reflecting an inability to generate or sustain an effective antitumor immune response, and acquired resistance, which emerges after an initial period of immune control [1,4]. Despite its usefulness, this binary classification does not capture the full spectrum of clinical presentations, which also includes early progression, mixed or dissociated responses, isolated progression, and relapse after prolonged disease control [1,5,6].

There are several, often overlapping, mechanisms of immunotherapy resistance. Tumors may evade immune surveillance through loss of antigen presentation, altered interferon signaling, immune-excluded oncogenic programs, dedifferentiation, or promotion of an immunosuppressive tumor microenvironment (TME) [7,8]. Because these mechanisms differ, no single salvage approach suits all patients. Importantly, resistance biology should inform rather than dictate treatment selection. The extent and tempo of progression, anatomical site, prior therapies, patient fitness, and available clinical evidence first determine the clinically appropriate treatment options, while characterization of the resistance mechanism may help refine the choice among these options when an actionable pathway can be identified. Isolated progression manifested by a small number of cancer sites, typically 1 to 5 lesions, is referred to as oligoprogression and may be managed with local therapy in selected cases, whereas broader systemic resistance may require mechanism-matched combinations rather than simply intensifying checkpoint blockade [6]. Accordingly, in selected clinically stable patients with oligoprogression or mixed/dissociated responses, continuing ICI while treating the discordant lesions with local therapy may be considered, particularly when most disease sites remain controlled [6,9]. The pattern of progression may further help identify patients suitable for this approach. In a retrospective melanoma cohort, patients with progression due to growth of established metastases had substantially longer progression-free survival after local therapy than those who developed new metastatic lesions, suggesting that progression confined to previously recognized disease may identify a subgroup more likely to achieve durable control with local treatment [10].

Trial outcomes can also overstate how well ICIs perform in routine practice. Patients with brain metastases, poor performance status, autoimmune disease, or organ dysfunction are often underrepresented in pivotal trials. In the Dutch Melanoma Treatment Registry, about 40% of systemically treated patients would have been ineligible for phase III trials, and their median overall survival (OS) was 8.8 months compared with 23 months among trial-eligible patients [11]. In a U.S. National Cancer Database analysis, only 36% of patients with metastatic melanoma received first-line immunotherapy, with use shaped by access, comorbidity, and sociodemographic factors [12]. Negative trials can therefore be as informative as positive ones, as they can help better understand whether poor outcomes reflect tumor biology, patient selection, endpoint choice, or weak mechanistic matching [13].

**Figure 1:**
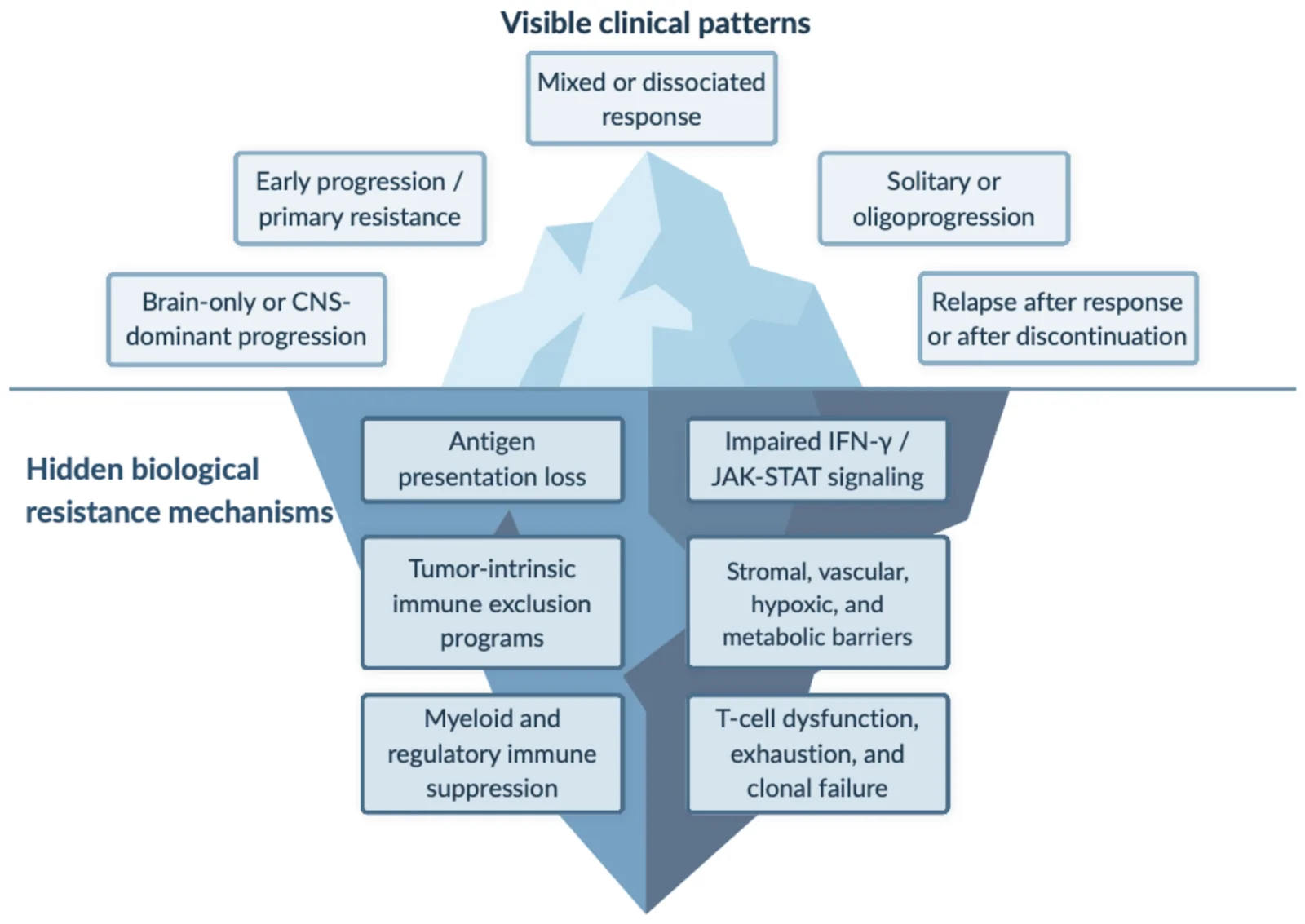
Visible and hidden layers of melanoma immunotherapy failure.

In this review, we examine immunotherapy resistance in melanoma as a clinically meaningful endpoint. Rather than considering resistance mechanisms in isolation, we integrate the clinical pattern of treatment failure with the processes that may underlie it, the limitations of available biomarkers, and lessons from unsuccessful therapeutic trials. This review helps link the visible phenotype of progression to candidate resistance biology and then to the therapeutic and trial-design questions that follow. This approach is summarized in Figure 1, which frames melanoma ICI resistance as visible patterns of progression arising from overlapping hidden resistance mechanisms.

## 2. Methods

This narrative review is based on a search of PubMed/MEDLINE, Web of Science, and ClinicalTrials.gov for publications from January 2010 to May 2026. The year 2010 was selected as a pragmatic starting point because it marked the emergence of modern immune checkpoint blockade as an effective therapeutic strategy in advanced melanoma, including the first phase III evidence of improved overall survival with ipilimumab. Earlier publications were included when they provided foundational clinical or mechanistic concepts necessary to interpret the contemporary literature, such as established response-assessment frameworks or seminal studies of tumor–immune interactions relevant to immunotherapy resistance. The following search terms were used in different combinations: “melanoma,” “immune checkpoint inhibitors,” “anti-PD-1,” “anti-CTLA-4,” “primary resistance,” “acquired resistance,” “pseudoprogression,” “hyperprogression,” “tumor microenvironment,” “antigen presentation,” “interferon signaling,” “myeloid-derived suppressor cells,” “tumor-associated macrophages,” “T-cell exhaustion,” “biomarkers,” “ctDNA,” and “clinical trials.” Studies were selected based on clinical relevance, mechanistic contribution, inclusion of melanoma-specific data, relevance to resistance patterns, response assessment, biomarker limitations, or trial design. Preference was given to peer-reviewed original studies, translational melanoma studies, consensus statements, pivotal trials, and reviews that were directly relevant to the clinical or mechanistic questions addressed in this article. ClinicalTrials.gov was searched to identify relevant melanoma immunotherapy trials and to verify trial design and status. Trial efficacy and safety findings were incorporated when supported by peer-reviewed publications; ongoing or terminated trials without available published outcome data were not used to support conclusions regarding therapeutic efficacy. Reference lists of selected articles were manually screened for additional relevant studies. Given the narrative and non-systematic design of this review, formal quality assessment, meta-analysis, and protocol registration were not performed. Its conclusions should be viewed as a thematic synthesis of the available literature rather than a systematic evidence estimate.

## 3. Results

### 3.1. Visible Clinical Patterns of Immunotherapy Failure

#### 3.1.1. Primary and Acquired Resistance

Clinical resistance to checkpoint blockade is often classified as primary or acquired, a distinction that carries prognostic, therapeutic, and trial-interpretation weight. According to the Society for Immunotherapy of Cancer (SITC), primary resistance refers to progressive disease or stable disease lasting less than six months that occurs following at least two cycles (approximately 6–12 weeks) of anti–PD-1 therapy exposure [4]. Secondary or acquired resistance refers to progressive disease that occurs after an initial complete or partial response or after stable disease lasting at least 6 months, following at least 6 months of treatment exposure [1,4].

However, this binary classification does not adequately reflect the clinical heterogeneity of melanoma progression during or after immunotherapy. Resistance may present as rapid early progression, mixed or dissociated response, oligoprogression, central nervous system progression, or relapse after elective treatment discontinuation. These phenotypes describe a different dimension of treatment failure from the conventional primary/acquired classification. Primary and acquired resistance are principally defined by the timing of progression and whether meaningful disease control was previously achieved, whereas the additional phenotypes describe the distribution, kinetics, or anatomical pattern of progression. Early progression refers to failure occurring near the first planned disease assessment; mixed or dissociated response describes simultaneous regression or stability of some lesions with progression of others; oligoprogression refers to progression at a limited number of disease sites while the remaining disease remains controlled; and CNS progression refers to progression within the brain or leptomeningeal compartment, sometimes despite extracranial disease control. Relapse after treatment discontinuation represents recurrence following cessation of therapy after an initial period of disease control. These categories are therefore complementary rather than mutually exclusive; for example, oligoprogression or CNS-dominant progression may occur in a patient with acquired resistance.

In a multicenter cohort of 299 metastatic melanoma patients who achieved complete or partial responses on PD-1-based therapy and later progressed, median time to acquired resistance was 12.6 months, and progression was limited to a single organ in 65% and to a solitary lesion in 51% of cases [1]. This heterogeneity in tumor progression supports subdividing acquired resistance into solitary progression, single-site oligoprogression, and multisite systemic relapse, each with distinct salvage options and prognostic implications.

#### 3.1.2. Early Progression and Hyperprogression

Beyond the primary/acquired dichotomy, the timing of progression is important. Early progression is common: in the Food and Drug Administration (FDA) pooled analysis of anti–PD-1 trials in melanoma, about two-thirds of progressions defined by the Response Evaluation Criteria in Solid Tumors (RECIST) occurred at the first disease assessment [9]. The first planned assessment, usually at 8–12 weeks, is therefore important because it could guide subsequent management [9].

Within this early window, rapid clinical deterioration may raise concern for hyperprogressive disease, but this should be distinguished from rapid disease progression caused by primary resistance. Hyperprogression refers to acceleration of tumor growth kinetics after ICI exposure, although inconsistent definitions limit incidence estimates [14]. In melanoma, true hyperprogression appears uncommon: in one cohort of 206 treatment episodes, only 1 of 75 evaluable episodes met stringent criteria [15]. Most early progression in melanoma should therefore be interpreted as aggressive primary resistance, unless tumor-growth acceleration is objectively documented after initial treatment [14,15].

#### 3.1.3. Mixed and Dissociated Responses

Responses to checkpoint blockade are not always uniform across lesions within the same patient at a given time. Although conventional guidelines classify such patients as having progressive disease, the terms “dissociated” or “mixed response” are more accurate [5].

A progressing lesion is not necessarily “immunologically cold,” and switching systemic treatment is not always the best response. Progressing lesions in patients with dissociated responses can exhibit features of active immune engagement, including increased CD8+ T-cell infiltration and interferon gamma (IFN-γ) gene expression, as well as PD-L1 expression comparable to that of responding lesions from the same patient, indicating that immune infiltration alone does not guarantee tumor control [16]. Stronger T-cell activation and effector-memory programs correlate with improved progression-free survival (PFS) at the cohort level [17], suggesting that a local immune response may be insufficient once tumors acquire intrinsic escape mechanisms [5].

#### 3.1.4. Relapse After Response and Treatment Discontinuation

Relapse after an initial response may occur either during ongoing anti–PD-1 therapy or after treatment discontinuation. Anatomically, acquired resistance may involve new lesions (45%), prior lesions (35%), or a combination of both (20%), most commonly involving the lymph nodes and brain [1]. The timing of relapse after the last treatment dose is not uniform. In the adjuvant setting, relapse occurring less than 12 weeks after the last dose is considered early, whereas relapse occurring at least 12 weeks after the last dose is considered late [4].

Relapse may also occur after elective treatment discontinuation. In KEYNOTE-001, complete responders who discontinued pembrolizumab had an estimated 24-month disease-free survival of approximately 90%, with stepwise declines for partial responders and stable disease [2,3]. Real-world data similarly suggest that disease control can remain durable after elective discontinuation, although outcomes vary according to the depth of response at the time treatment is stopped [3].

#### 3.1.5. Central Nervous System Progression

Central nervous system (CNS) involvement, including leptomeningeal and brain metastases, can behave differently from extracranial disease during anti–PD-1 therapy, progressing despite control at other sites or developing de novo in patients whose extracranial disease is otherwise responding. In one cohort, the brain was a common site of relapse, with both solitary and multifocal progression [1]. A dedicated magnetic resonance imaging (MRI) surveillance study highlighted the risk of CNS progression [18]. In that study, 24% of patients without brain involvement developed brain metastasis within two years [18]. Among patients with established brain metastases, findings on surveillance MRI led to a change in treatment strategy after 45% of scans [18]. The distinct brain microenvironment, reduced T-cell infiltration, distinct myeloid populations, and impaired antigen presentation likely limit ICI efficacy even when extracranial disease remains controlled [19,20]. Here, surveillance becomes part of resistance prevention rather than mere documentation of progression.

### 3.2. Response Assessment and Potential Reporting Pitfalls

#### 3.2.1. Imaging Pitfalls

The evaluation of ICI efficacy in melanoma is more challenging than cytotoxic chemotherapy, primarily because radiologic changes can be misinterpreted and assessments mistimed, leading to premature treatment discontinuation. Initial response assessments may be difficult to interpret because early radiographic changes do not always reflect true tumor progression. Formal response criteria may further complicate this problem. Because RECIST 1.1 relies on anatomic size and can score progression from a single lesion, patients with overall tumor shrinkage or stable disease may be labeled as having progressive disease [21,22]. The immune-adapted iRECIST criteria were developed to reduce this risk, although they remain limited by their reliance on anatomic tumor size [23]. Integrating metabolic imaging (FDG-PET) or circulating tumor DNA (ctDNA) kinetics may help overcome the limitations of size-based assessment [24].

#### 3.2.2. Pseudoprogression

A key challenge in melanoma assessment and management is pseudoprogression, which is an initial radiographic increase in tumor size or the emergence of new lesions, followed by a decrease in total tumor burden. It is generally attributed to immune-cell infiltration, inflammatory changes, and localized edema within the tumor microenvironment (TME) that mimic growth rather than reflect true progression [25]. In a systematic review and meta-analysis of ICI-treated solid tumors, the pooled incidence of pseudoprogression was 6.0% overall and 6.4% in melanoma [26].

Because of this phenomenon, clinically stable patients require confirmatory assessment rather than immediate treatment cessation. iRECIST classifies these cases as unconfirmed progressive disease (iUPD), requiring a follow-up scan 4 to 8 weeks later to confirm true progression (iCPD) before treatment is stopped. In one melanoma cohort, 33.6% of patients initially classified as having progressive disease did not meet criteria for confirmed progression on follow-up [23]. Patients with melanoma with pseudoprogression have demonstrated longer median OS than those with true uncontrolled disease (29.9 months versus 8.0 months), underscoring the risk of premature discontinuation based on initial imaging alone [27]. Nevertheless, because pseudoprogression is uncommon, treatment beyond initial progression should be reserved for clinically stable patients without rapid decline, symptomatic progression, or imminent organ compromise.

#### 3.2.3. Endpoint Interpretation in Clinical Response Assessment

Trial interpretation is further complicated by endpoint selection, because conventional metrics such as early PFS and objective response rate (ORR) may not fully capture the durable remissions seen in selected melanoma patients [28]. RECIST 1.1 may underestimate the therapeutic benefit in approximately 15% of pembrolizumab-treated patients by classifying “atypical responders” as treatment failures [22]. This may explain why ORR may correlate moderately with PFS but has a weaker correlation with OS in melanoma. In recent ICI trials, the correlation between median PFS and OS was not statistically significant (R = 0.45, *p* = 0.11), likely due to delayed immune activation, pseudoprogression, and post-progression treatment [28].

More reliable interpretation of ICI trials may require the use of immune-specific and time-dependent endpoints. KEYNOTE-001 highlights this: patients with discordant responses (progressive disease by RECIST but nonprogressive by immune criteria) achieved a 2-year OS rate of 37.5%, versus 17.3% among true progressors [22]. Measures such as survival, duration of response, restricted mean survival time, confirmatory imaging rules, and clear criteria for treatment beyond progression may better reflect durable clinical benefit that is not captured by early radiographic changes alone.

### 3.3. Tumor-Intrinsic Mechanisms of Resistance

#### 3.3.1. Loss of Antigenicity

Melanoma is one of the most immunogenic human malignancies due to its high mutational burden and the resulting production of neoantigens, which partly explain its sensitivity to checkpoint blockade [7]. However, under sustained immune pressure, this immunogenicity can erode. As cytotoxic T cells eliminate clones expressing highly immunogenic neoantigens, less recognizable tumor populations outcompete them, while neoantigen-rich clones decline as resistance emerges [7,29]. In a patient with relapsed melanoma following pembrolizumab, Zaretsky and colleagues identified a truncating β2-microglobulin (B2M) mutation that abolished major histocompatibility complex (MHC) class I surface expression; other resistance-associated mutations were subsequently reported in other cohorts [8,30]. Melanoma cells can also dedifferentiate into a resistant state, particularly after inflammation, shifting toward a neural crest-like or undifferentiated state and losing lineage-specific melanocytic markers [31]. This switch can occur without new mutations and can be reversible. When tumor cells lose the lineage-specific antigens the immune system recognizes, simply intensifying checkpoint blockade may not restore effective recognition [32].

#### 3.3.2. Interferon Signaling Defects

Resistance can also occur while the cancer cells remain “visible” to the immune system, through impaired responsiveness to effector signals. IFN-γ released by activated T cells normally activates Janus kinase 1/2 (JAK1/JAK2)-signal transducer and activator of transcription 1 (STAT1) signaling, which increases MHC class I expression, recruits effector cells through CXCL9 and CXCL10, and enhances tumor susceptibility to cytotoxic killing [33]. The same study that identified B2M loss also found acquired loss-of-function JAK1 and JAK2 mutations in resistant lesions despite continued T-cell infiltration of the cancers [8]. Impaired JAK/STAT signaling can also result from transcriptional silencing or adaptive rewiring. However, a persistent IFN-responsive transcriptional program is strongly associated with clinical response in multiple melanoma cohorts [34,35]. While interferons support protective immunity by enhancing antigen presentation, sustained expression can also lead to inhibitory-ligand expression and effector-cell exhaustion [36]. Effective immunity requires sufficient interferon responsiveness that supports antigen presentation and effector recruitment without driving the tumor microenvironment toward adaptive resistance [37].

#### 3.3.3. Oncogenic Immune Evasion Pathways

Tumor-intrinsic signaling is not only permissive for immune evasion, but can also actively promote it [38]. The mitogen-activated protein kinase (MAPK) pathway is activated in the majority of melanomas, mainly through BRAF mutations, which occur in approximately half of cutaneous melanomas, and NRAS mutations, which are found in around 15–25% of non-uveal melanomas [39,40]. In addition to promoting melanoma-cell proliferation, MAPK signaling can reduce immune recognition by decreasing expression of differentiation antigens and pro-inflammatory chemokines while increasing immunosuppressive cytokines [41]. Acutely inhibiting the MAPK pathway can rapidly reverse many of these effects by increasing antigen expression and CD8+ T-cell infiltration [41]. However, this effect may be transient, as prolonged inhibition can reduce antigen presentation, impair CD8+ T-cell function, and promote features of T-cell exhaustion [42]. Other pathways limit immunity by different mechanisms. Activation of the tumor-intrinsic WNT/β-catenin pathway prevents BATF3-lineage dendritic cells from reaching the tumor, creating immune-desert lesions in which effective T-cell priming cannot occur despite checkpoint blockade [43]. PTEN loss may also reduce T-cell infiltration and cytotoxic activity [44]. Together, these findings show that melanoma cells can establish immune resistance before or independently of effective T-cell infiltration.

#### 3.3.4. Targeted Therapy–Immune Resistance Links

Resistance to targeted therapy and to immunotherapy are biologically related rather than mutually exclusive. During prolonged BRAF/MEK inhibition, melanoma can adopt dedifferentiated, immune-evasive states with reduced antigen presentation, impaired T-cell activity, and features that may influence subsequent checkpoint blockade [42,45]. Consistent with this, the DREAMseq (Doublet, Randomized Evaluation in Advanced Melanoma Sequencing) trial showed better survival when nivolumab plus ipilimumab preceded BRAF/MEK inhibition, and the SECOMBIT (Sequential Combo Immunotherapy and Targeted Therapy) trial provided additional support for introducing immunotherapy early in the treatment sequence [46,47]. Targeted therapy remains an important option for patients in need of rapid disease control, although prolonged first-line BRAF/MEK inhibition may lead to states that are less responsive to subsequent immunotherapy. The major challenge in this rapidly evolving field is to identify treatment sequences that preserve immune sensitivity.

### 3.4. Tumor Microenvironment and Immune Exclusion

#### 3.4.1. Immune-Inflamed, Immune-Excluded, and Immune-Desert Phenotypes

Response rates to immune checkpoint blockade (ICB) vary greatly across tumor types [48], in part because of differences in the tumor immune microenvironment (TIME) [49].

Early classifications divided tumors based on the density of tumor-infiltrating lymphocytes (TILs), particularly CD8+ T cells, distinguishing tumors with abundant from those with limited immune infiltration [50]. Later studies further classified tumors into three phenotypes: immune-inflamed tumors, immune-excluded tumors, or immune-desert tumors [51]. In melanoma, Młyńska SUPP. identified these subtypes using gene-expression and histologic features, with inflamed tumors showing more favorable survival outcomes than non-inflamed tumors [52]. This may reflect greater tumor antigenicity and mutational burden, together with IFN-γ-producing CD8+ T-cell infiltration and preexisting antitumor immunity [53].

#### 3.4.2. Regulatory T-Cell-Mediated Suppression

Among the cellular mechanisms that contribute to immunosuppression, regulatory T cells (Tregs) are especially important [54]. Tregs are found in many tumors, including melanoma, and their presence is associated with resistance to ICIs [55]. Their recruitment and activation are promoted by inflammatory mediators, altered antigen presentation, and immunoregulatory dendritic-cell states [56]. Additionally, tumor-associated macrophages (TAMs) can further drive differentiation of CD4+ T cells into Tregs by secreting interleukin-10 (IL-10) and transforming growth factor-β (TGF-β) [57].

The balance between cytotoxic T lymphocytes and Tregs is an important marker of the TIME phenotype, with higher cytotoxic-to-Treg ratios generally seen in inflamed tumors [58]. Tregs suppress effector T cells through inhibitory cytokines, such as IL-10, TGF-β, and IL-35 [59,60].

Immune checkpoint pathways also modulate Treg activity. PD-1/PD-L1 blockade may enhance Treg proliferation or function in some contexts [61], whereas anti-CTLA-4 antibodies can deplete intratumoral Tregs through Fcγ receptor-expressing macrophages, potentially contributing to their efficacy in melanoma models [62].

Melanoma-associated Treg populations that co-express surface markers such as CD39, PD-1, or CCR8 represent highly suppressive subsets and have been linked to poor prognosis and resistance to ICB [63].

#### 3.4.3. Stromal and Vascular Barriers

The structural properties of tumors also contribute to resistance to immunotherapy. Pressure from rapid cell proliferation and increased ECM production can prevent lymphocytes from infiltrating into tumors, leading to immune evasion, particularly in the tumor center [64]. Cancer-associated fibroblasts (CAFs) are central to this process, depositing and organizing collagen and fibronectin, increasing matrix tension, compressing adjacent blood vessels, and physically marginalizing T cells [65,66].

In addition to acting as a physical barrier, the ECM can influence immune function. Dense collagen may reduce cytokine and chemokine diffusion, redirect T-cell migration along collagen fibers rather than toward tumor cells, and suppress cytotoxic activity through collagen-LAIR-1 signaling [67,68,69].

Disturbed tumor endothelium likewise acts as a barrier for immune-cell entry. Disorganized vessels reduce lymphocyte adhesion, extravasation, and infiltration, partly through low adhesion-molecule expression [70]. Vascular endothelial growth factor (VEGF) promotes immune suppression, while VEGF blockade can normalize tumor vessels and increase T-cell infiltration [71].

The blood–brain barrier (BBB) represents an additional vascular challenge in melanoma brain metastases. Its specialized endothelial architecture restricts the entry of circulating therapeutics, while metastatic growth can disrupt the BBB and produce a heterogeneous blood–tumor barrier with nonuniform permeability, resulting in variable drug exposure within brain lesions [72]. The BBB also influences immune-cell entry into the CNS; however, activated T cells can traffic to melanoma brain metastases, and preclinical data show increased CD8+ T-cell recruitment following combined PD-1/CTLA-4 blockade [73]. Thus, the BBB may contribute to the distinct therapeutic behavior of brain metastases but does not constitute an absolute barrier to checkpoint immunotherapy, as clinically meaningful intracranial responses have been observed [19].

#### 3.4.4. Hypoxia and Metabolic Constraints

Melanoma cells can exploit normal metabolic function to promote proliferation and survival [74]. Rapidly dividing cancer cells create a hypoxic, lactate-rich, acidic, and amino acid-depleted microenvironment that impairs intratumoral T-cells [74]. Hypoxia is particularly important. The tumor’s unusual vasculature creates an oxygen gradient with a hypoxic core [75], confining T cells to perivascular regions while immunosuppressive cells populate the core. In preclinical models including B16 melanoma, hypoxia reduced IFN-γ-mediated MHC class I induction and impaired CD8+ T-cell proliferation and IFN-γ production, weakening antigen presentation and tumor-cell killing [76].

Metabolic competition further contributes to resistance. Lactate produced by highly glycolytic tumors, including melanoma models, promotes PD-1 expression in Tregs, enhancing their suppressive activity and reducing the efficacy of PD-1 blockade [77].

Amino acid depletion adds an additional layer: depletion of arginine by tumor cells impairs T-cell function [78], while tryptophan catabolism through indoleamine 2,3-dioxygenase 1 (IDO) or tryptophan 2,3-dioxygenase (TDO) generates kynurenine, activates aryl hydrocarbon receptor (AhR) signaling, promotes Treg differentiation, and suppresses antitumor immunity [79]. Together, these findings suggest that melanoma resistance is caused by local metabolic conditions that prevent T cells from recognizing, surviving within, and eliminating tumor cells, in addition to antigen loss or checkpoint signaling.

### 3.5. Myeloid and Macrophage Suppression

The different barriers described in Section 3.4 are shaped by tumor cells, fibroblasts, endothelial cells, and tumor-educated myeloid populations. Among these, myeloid-derived suppressor cells (MDSCs) and tumor-associated macrophages (TAMs) are especially important, because they combine metabolic suppression, cytokine-mediated inhibition, checkpoint-ligand expression, and remodeling of the microenvironment.

#### 3.5.1. Myeloid-Derived Suppressor Cells (MDSCs)

MDSCs are immature myeloid cells whose differentiation is arrested by persistent tumor-associated inflammation [80]. In humans, they are broadly divided into monocytic (M-MDSC) and polymorphonuclear (PMN-MDSC) subsets [80]. Their accumulation in melanoma is actively promoted by tumor-derived GM-CSF, G-CSF, IL-6, IL-1β, VEGF, and PGE2 [81] and their trafficking is subset specific: M-MDSCs are recruited mainly through CCR2 and CCR5, and PMN-MDSCs through CXCR1/2 [80,82].

Within the tumor, MDSCs produce many of the metabolic barriers discussed in Section 3.4: they deplete arginine through arginase-1 (ARG1), catabolize tryptophan through IDO, produce nitric oxide and reactive oxygen species that impair T-cell receptor (TCR) signaling, convert extracellular ATP to adenosine through CD39/CD73, and express high levels of PD-L1 [81]. Because these mechanisms occur within the same cell population, blocking any one pathway is unlikely to fully restore antitumor immunity. Tumor-intrinsic IDO further reinforces this cycle. Holmgaard and colleagues showed that forced IDO expression in melanoma recruits and activates MDSCs through a Treg-dependent mechanism, an effect reversed by IDO inhibition [83]. Thus, MDSCs are not simply a separate suppressive pathway, but a downstream amplifier of tumor-intrinsic immune escape.

Clinically, MDSC levels are associated with checkpoint-inhibitor resistance. In patients with metastatic melanoma treated with anti-PD-1 therapy or combination ICIs, non-responders had higher baseline PMN-MDSC and M-MDSC frequencies, preserved suppressive activity against autologous T cells, and increased plasma IL-6 and IL-8 levels, features absent in responders [84]. Together, MDSC frequency, suppressive function, and inflammatory mediators such as IL-6 and IL-8 may constitute a more informative suppressive myeloid profile than any single measure alone [80,84].

#### 3.5.2. Tumor-Associated Macrophages (TAMs)

In contrast to MDSCs, TAMs are mature myeloid cells educated by the tumor and often comprise a large fraction of the melanoma immune infiltrate [65,85]. They derive from tissue-resident macrophages residing in skin and from circulating monocytes recruited through CCL2–CCR2 signaling and reprogrammed locally by tumor cues [85]. Although macrophages are usually described by the M1/M2 dichotomy, melanoma TAMs are better viewed as a continuum whose position is determined by the local signaling environment [85,86]. Early lesions may contain M1-like tumoricidal macrophages, whereas advanced melanoma is commonly enriched for M2-like programs driven by hypoxia, CSF-1, IL-10, TGF-β, PGE2, tumor-derived exosomes, and chronic inflammation [67,85,87].

Their suppressive output extends further than that of MDSCs [85,86]. TAMs uniquely remodel tumor architecture, producing VEGF, IL-6, and matrix metalloproteinases that drive aberrant vascular remodeling, ECM degradation, and tumor dissemination [88]. TAMs can also promote melanoma phenotype switching through factors such as TGF-β, IL-6, and IL-8, favoring more invasive, therapy-resistant states [85].

#### 3.5.3. Macrophage Reprogramming Strategies

Given this plasticity, therapeutic strategies have shifted from macrophage depletion toward functional reprogramming of the myeloid compartment [87,89], on the premise that restoring pro-inflammatory, antigen-presenting, and T-cell-supportive macrophage functions is more effective than broadly eliminating macrophages [87,89]. Investigational approaches to macrophage reprogramming include blocking monocyte and TAM recruitment through CCL2-CCR2, CSF-1-CSF-1R, and C5a-C5aR signaling; enhancing phagocytosis through CD47-SIRPα blockade; and promoting M1-like polarization through TLR agonists, CD40 agonists, PI3Kγ inhibition, CSF-1R modulation, and metabolic or epigenetic interventions [90,91,92].

This therapeutic logic is supported by melanoma models in which macrophage reprogramming improved immune infiltration and strengthened checkpoint blockade responses [93,94]. Targeting the scavenger receptor MARCO (Macrophage Receptor with Collagenous Structure) is one example. Combined with CTLA-4 inhibition, MARCO blockade enhanced tumor regression by inducing M1-pattern chemokines, increasing immune-cell infiltration, and recruiting mature conventional dendritic cells [93]. Importantly, macrophage depletion abolished this effect, suggesting that the therapeutic effect depended on using macrophages functionally rather than removing them [93].

Inhibition of macrophage migration inhibitory factor (MIF) is another example [94]. In resistant melanoma models, blocking the MIF-CD74 axis increased CD8+ T-cell infiltration, promoted M1-like macrophage conversion, reduced lactate production, and decreased HIF-1α and PD-L1 expression, linking macrophage reprogramming to both immune and metabolic remodeling [94]. These examples support TAM reprogramming as a means of converting suppressive myeloid niches into immune-permissive ones, though clinical translation will require patient selection and pharmacodynamic confirmation rather than broad empiric use [93,94,95].

### 3.6. Clonal Dynamics and T-Cell Failure

#### 3.6.1. Clonal Expansion and Collapse

Resistance to immune checkpoint blockade does not necessarily indicate absence of T cells within melanoma lesions. T cells can be present in a tumor yet non-reactive, exhausted, or fail to persist following expansion [38,96]. Durable response requires melanoma-reactive clones that recognize tumor antigen, expand after therapy, retain cytotoxic function, and survive antigenic pressure [97,98]. This clonal layer is distinct from tumor-intrinsic escape, immune exclusion, and myeloid suppression [38,99].

A key question is whether response depends on revival of preexisting intratumoral clones or recruitment of new peripheral clones [96,97]. Single-cell and TCR sequencing studies suggest that response often depends on expansion of preexisting tumor-reactive CD8+ clones, while non-response may involve ineffective replacement by peripheral or bystander clonotypes [96]. This explains why an inflamed lesion can still progress: immune infiltration may be abundant but functionally mismatched to the tumor [96,98]. In adoptive cell therapy, poor response can similarly occur when infused or intratumoral T-cell pools contain many non-tumor-reactive bystander clones [98].

Checkpoint agents also shape clonal behavior differently. Anti-CTLA-4 can broaden or mobilize the peripheral repertoire, while anti-PD-1 acts more directly on exhausted tumor-reactive clones within the tumor microenvironment [100,101]. Combination therapy may generate waves of clonal expansion, beginning with progenitor exhausted populations and progressing toward cytotoxic effector states [100]. The ability to expand and reinvigorate effective CD8 T-cell clones appears to differ between responders and nonresponders [96,100], suggesting that resistance may reflect failure to generate, reinvigorate, or maintain an effective tumor-reactive clonal response.

#### 3.6.2. TCR Diversity Loss

Analysis of the T-cell receptor (TCR) repertoire in tumor-infiltrating T cells provides insights into the quality of antitumor immunity. However, higher diversity or even increased clonality does not necessarily translate into stronger antitumor immunity [98,102]. Tumors can contain expanded T-cell populations unrelated to melanoma recognition, including virus-specific or inflammatory bystander clones recruited by chemokine gradients [103]. Most parameters that describe the diversity of a repertoire, like Shannon entropy, clonality, and D50 (the number of dominant clones accounting for half of all reads), do not distinguish tumor-reactive clones from non-reactive clones [98,102].

More informative approaches assess whether expanded clones show tumor enrichment, shared sequence features, antigen-driven selection, or functional reactivity [102]. TCR convergence is useful here, because different nucleotide sequences can encode similar or identical CDR3 amino acid motifs, suggesting selection by a shared antigen rather than random expansion [102]. Thus, the key question is whether the repertoire contains tumor-reactive clones capable of sustained effector function, not simply whether it is broad or narrow [98,102].

Functional repertoire diversity may also decline during acquired resistance. Initial expansion of tumor-reactive clones can result in regression of the tumor and then, over time, due to chronic antigen exposure, terminal exhaustion, and tumor immunoediting, selection against the effective clones can occur [104]. Hence, the resulting pool of T cells may become dominated by exhausted, nonreactive, or bystander populations [103,104].

#### 3.6.3. Resistance Signaling Interactions

Tumor-intrinsic resistance mechanisms also destabilize T-cell clonal control. When melanoma loses antigen presentation, weakens interferon responsiveness, or adopts exclusionary transcriptional states, tumor-reactive clones may lose the signals required for recognition, persistence, and renewal [105,106]. In this sense, antigen loss, JAK/STAT defects, WNT/β-catenin activation, PTEN loss, and MAPK inhibitor-associated immune remodeling are not only tumor-cell programs; they are pressures that reshape the useful T-cell repertoire [44,45,107]. Even antigen-selected clones may lose their effectiveness when their target antigen is no longer presented or when the inflammatory signals needed to sustain them are lost [102,106]. During acquired resistance, previously effective T-cell clones may become less relevant as resistant melanoma subpopulations emerge [105,106]. Melanoma resistance often reflects a mismatch between T-cell quantity and T-cell quality: T cells may remain within the tumor, but the clones needed to maintain tumor control may be absent, dysfunctional, or unable to persist [103,104].

### 3.7. Biomarkers: Why Prediction Still Falls Short

If resistance in melanoma is biologically heterogeneous, biomarkers are needed to determine which mechanism predominates in a particular tumor. In practice, no single marker achieves this. Each is constrained by technical variability, temporal and spatial heterogeneity, or limited biological scope, and only captures one aspect of the tumor-immune interaction.

#### 3.7.1. PD-L1 Limits

PD-L1 expression remains one of the most widely studied biomarkers for immunotherapy responsiveness, but its clinical reliability is limited by both technical and biological factors. Discordant results are possible with different antibody clones, staining platforms and scoring systems, including tumor proportion score (TPS), combined positive score (CPS), and immune cell scoring [108,109]. The methodological differences lead to inconsistencies in PD-L1 positivity classification within labs and trials [110]. Moreover, different cut-off thresholds, such as 1%, 5%, or 50%, and differences in whether tumor cells or immune infiltrates are assessed, further reduce reproducibility [111,112].

PD-L1 expression is also dynamic. In melanoma, its expression can reflect adaptive immune resistance driven by local inflammatory signaling, rather than a stable property captured by one biopsy [113]. Therefore, a single biopsy may not accurately represent the tumor’s immunological state over time, and PD-L1 expression may differ between the primary tumor and metastatic lesions, limiting the reliability of one measurement across the full disease burden [111]. This is why PD-L1 may enrich for response in some contexts but remains insufficient as a stand-alone selector for melanoma immunotherapy [112]. Even in tumor types where PD-L1 is incorporated into treatment-selection criteria, its predictive utility is assay- and indication-specific rather than universally reliable [110]. In RELATIVITY-047, exploratory subgroup analyses showed a greater progression-free benefit with nivolumab–relatlimab over nivolumab in patients with PD-L1 expression <1% (HR 0.66) than in those with PD-L1 expression ≥1% (HR 0.95); however, these subgroup findings do not establish PD-L1 as a validated selector for nivolumab–relatlimab in melanoma [114].

#### 3.7.2. TMB Limits

Tumor mutational burden (TMB) is another commonly studied biomarker, defined as the number of somatic mutations per megabase of DNA, on the premise that higher mutation burden increases neoantigen generation, immunogenicity, and the probability of immune recognition [115]. However, its predictive value depends not only on mutation number but also on immunogenic quality [116]. Not all mutations produce neoantigens capable of activating T-cell responses, so tumors with high mutation counts may still fail to respond if antigen presentation, interferon signaling, T-cell infiltration, or effector function is impaired [117].

TMB measurement is also technically variable. Whole-exome sequencing and targeted panels assess different genomic regions, and differences in panel size, sequencing depth, bioinformatic pipelines, and mutation filtering criteria can substantially affect estimates [116,118].

A threshold of ≥10 mutations/Mb has been adopted for the FDA tumor-agnostic pembrolizumab indication and is therefore clinically relevant as a regulatory definition of TMB-high [119]. However, this threshold should not be regarded as a universal biological cutoff across tumor types because TMB estimates and their predictive value vary according to tumor context and testing methodology [116,119]. Therefore, TMB captures one dimension of tumor immunogenicity but does not fully reflect the functional immune competence required for checkpoint-inhibitor response [120].

#### 3.7.3. Spatial Heterogeneity

Solid tumors are heterogeneous ecosystems consisting of genetically and immunologically diverse subclones [111,121]. A biopsy taken from one region of the tumor may not reflect the entire tumor burden, particularly when PD-L1 expression, mutational profiles, and immune features differ between primary tumors and metastases or across metastatic lesions within the same patient [111]. For example, A single-lesion biopsy may incompletely represent the broader tumor-immune landscape because immune phenotypes and other microenvironmental features can differ between primary and metastatic lesions [118]. Multiregional sampling may improve accuracy, but it is costly, invasive, and not routinely feasible in clinical practice.

This limitation is particularly relevant in melanoma, where mixed responses, oligoprogression, and sanctuary-site progression suggest that different lesions evolve under different immune pressures. Therefore, biomarker measurement from one site may fail to capture the biology driving treatment response or resistance elsewhere. 

#### 3.7.4. Temporal Evolution

Biomarkers also change over time. Tumors evolve under therapeutic pressure, and treatments such as chemotherapy, radiotherapy, targeted therapy, or prior immunotherapy can modify tumor immunogenicity, immune infiltration, and checkpoint expression [7,42]. Therefore, biomarkers measured before treatment may no longer reflect the dominant biology later in the disease course, especially after resistant clones emerge.

Tumors also undergo natural longitudinal evolution through genomic instability and immune selection [121]. Clonal shifts alter mutation profiles, antigen presentation, and immune-evasion strategies. Because most biomarker assessments rely on single-time-point biopsies, they often fail to capture these changes and may poorly predict durable outcomes [121].

#### 3.7.5. Liquid Biopsy

Liquid biopsy, especially circulating tumor DNA (ctDNA) testing, offers a minimally invasive approach to monitoring tumor evolution in advanced melanoma [122]. Compared with tissue biopsy, ctDNA may capture genetic data from multiple tumor locations simultaneously, providing a comprehensive picture of tumor heterogeneity. It also permits repeated sampling during treatment, enabling dynamic monitoring of mutational burden, clonal evolution, and minimal residual disease [122].

Combined with radiologic imaging and other clinical parameters, ctDNA analysis may improve the prediction of the response, and molecular monitoring can sometimes detect changes in tumor burden earlier than traditional imaging, supporting adaptive treatment decisions [123]. However, liquid biopsy carries multiple limitations: ctDNA levels may be low in early-stage disease or minimal-shedding tumors, reducing sensitivity and risking false-negative results [124]. Therefore, ctDNA is best viewed as a complementary tool to tissue-based and radiologic assessment. While ctDNA-guided management has already become part of standard clinical practice in colorectal cancer, particularly for minimal residual disease assessment, its role in melanoma is rapidly expanding, with growing evidence supporting its utility for prognostication, treatment monitoring, and the early detection of relapse.

#### 3.7.6. Composite Models

Because individual biomarkers capture only part of the tumor–immune interaction, recent work has increasingly focused on composite predictive models. These integrate PD-L1 expression, TMB, gene-expression signatures, immune-infiltration patterns, ctDNA dynamics, and clinical features to better reflect the complexity of immunotherapy response [125,126,127]. Multi-omic approaches combining genomic, transcriptomic, and proteomic data may further improve accuracy by linking tumor antigenicity, immune activation, and resistance pathways within one framework [126,127].

Additional systemic biomarkers may also contribute to composite models. Gut microbiome composition has been associated with response to anti–PD-1 therapy in melanoma [128], and early studies of fecal microbiota transplantation in PD-1-refractory disease suggest that modifying the microbiome can influence antitumor immunity in a subset of patients [129]. Metabolomic profiling has likewise identified circulating metabolic patterns associated with response to checkpoint blockade [130], although neither microbiome nor metabolomic signatures are currently standardized or validated for routine treatment selection [128,130].

Clinical deployment remains challenging, however. Composite models require standardized assays, reproducible platforms, prospective validation, regulatory clarity, and interpretable data pipelines [131]. Without these, complex models may remain scientifically attractive but clinically difficult to implement. Artificial intelligence and machine learning approaches may facilitate this integration by combining high-dimensional molecular, imaging, pathological, and clinical data into composite predictive models; however, current applications in immuno-oncology remain predominantly retrospective and have not yet been validated for routine treatment selection [132]. The limited performance of individual biomarkers in melanoma does not mean prediction is impossible; rather, it shows that prediction must move beyond single static markers toward integrated, dynamic, and clinically validated models [126].

At present, there are no validated clinical criteria that reliably identify a single dominant resistance mechanism in an individual patient. Rather, a suspected resistance mechanism may be inferred by integrating the clinical pattern of progression with findings from the progressing lesion, when tissue is available, including genomic alterations, antigen-presentation status, immune-cell infiltration, and transcriptional or other molecular features. Serial tissue sampling and ctDNA may provide additional information on temporal evolution, but overlapping resistance pathways limit attribution to one mechanism. Thus, mechanism assignment currently remains probabilistic and investigational rather than a routine clinical classification, and prospective validation of integrated biomarker approaches is required. The major limitations and potential roles of current biomarkers and response-assessment tools in resistance-matched approaches are summarized in Table 1.

### 3.8. Lessons from Negative Trials

Negative trials in melanoma are useful because they identify where biological rationale, patient selection, biomarker enrichment, pharmacodynamic confirmation, and endpoint choice failed to align. This is especially important in immunotherapy-resistant melanoma, where the same clinical label, such as post-PD-1 progression, may include biologically distinct states including antigen presentation loss, immune exclusion, myeloid suppression, metabolic resistance, and terminal T-cell dysfunction.

#### 3.8.1. Failed Immune Intensification: Activation Is Not Enough

The clearest modern example is IDO1 (Indoleamine 2,3-dioxygenase 1) inhibition. In ECHO-301, epacadostat plus pembrolizumab did not improve progression-free survival or overall survival compared with pembrolizumab plus placebo in unresectable or metastatic melanoma [133]. This result was important because IDO1 inhibition was biologically plausible and supported by early-phase enthusiasm, yet failed in a large randomized trial. The lesson is not that IDO biology is irrelevant, but that pathway plausibility and early clinical signals are insufficient without clear target engagement, appropriate dosing, and enrichment for tumors in which the pathway is dominant.

Vaccine-based strategies point the same way. In metastatic melanoma, ipilimumab improved survival compared with gp100 peptide vaccine alone, but adding gp100 to ipilimumab did not improve survival over ipilimumab alone [134]. Similarly, Canvaxin plus BCG failed to improve survival after complete resection of stage IV melanoma [135], and although gp100 vaccine plus high-dose IL-2 improved response rate and progression-free survival, the overall survival benefit was not definitive [136]. Increasing antigen exposure or short-term immune activation does not reliably produce durable survival benefit.

Attempts to stimulate innate immunity have not been able to reach success to date. In ILLUMINATE-301, adding intratumoral tilsotolimod, a TLR9 agonist, to ipilimumab did not improve objective response rate or overall survival compared with ipilimumab alone in patients with anti-PD-1–refractory advanced melanoma [137].

Cytokine-based immune stimulation has faced the same problem. In PIVOT IO-001, bempegaldesleukin plus nivolumab produced a lower objective response rate, no progression-free survival benefit, no overall survival benefit, and more grade 3 to 4 or serious treatment-related adverse events than nivolumab alone [138]. Immune activation is not automatically therapeutic: if stimulation is not directed at the relevant resistant state, it may add toxicity without improving tumor control.

#### 3.8.2. Translational and Biomarker Mismatch: Pathway Presence Is Not Pathway Dependence

Many disappointing strategies fail because experimental systems do not reproduce the full resistance biology seen in patients. Preclinical models may show immune activation, increased T-cell infiltration, or reversal of a suppressive pathway, yet fail to capture lesion-level heterogeneity, prior treatment exposure, exhausted T-cell states, stromal exclusion, myeloid suppression, and adaptive upregulation of alternative checkpoints [139]. Therefore, preclinical immune activation should be treated as hypothesis-generating rather than proof that a combination will improve survival in an unselected population. 

ECHO-301 also illustrates a biomarker problem. Epacadostat was tested without requiring IDO1 expression, IDO pathway activation, or dependence on IDO-mediated immune suppression as an entry criterion [133]. As a result, any benefit in a biologically susceptible subgroup could have been diluted by enrolling patients whose tumors were not meaningfully driven by that pathway. A pathway may be present, measurable, and plausible, but still fail clinically if it is not the dominant mechanism of resistance in the treated population.

Exploratory biomarker findings identified after a trial should not be mistaken for clinical utility unless prospectively validated [127]. PD-L1 immunohistochemistry illustrates the broader problem because variability across antibody clones, staining platforms, scoring systems, cutoffs, and interpretation can misclassify patients and weaken enrichment [108,109,110]. Such limitations do not make biomarkers unimportant; they show that biomarker strategies must be prospectively planned, technically standardized, and biologically linked to the mechanism being targeted.

#### 3.8.3. Trial and Endpoint Mismatch: Unselected Resistance Is Not One Disease

Across ECHO-301 and PIVOT IO-001, one lesson is clear: suboptimal trial design can hide a beneficial effect when the population is too broad or target engagement undefined [133,140,141]. This is particularly important in post-PD-1 melanoma, where resistance can present as primary progression, delayed acquired resistance, oligoprogression, mixed response, brain progression, or relapse after discontinuation [4]. Because these patterns may reflect biologically distinct states, grouping them under one resistance label can dilute therapeutic signals [1,4,5].

Future trials should therefore begin by defining the resistance phenotype that the intervention is intended to address. A drug designed to reverse immune exclusion should not be tested the same way as one intended to restore antigen presentation, suppress myeloid-mediated inhibition, or reinvigorate exhausted T-cell clones. Trials should also include planned pharmacodynamic confirmation and biomarker analyses. Adaptive multi-arm designs may be especially useful in post-PD-1 populations because they can test several strategies simultaneously, stop ineffective arms early, and focus recruitment on more promising approaches [141,142,143]. Eligibility must be balanced since narrow enrollment limits generalizability, while broad criteria risk diluting mechanism-specific effects.

Endpoint selection can compound this problem. As discussed earlier, response rate and progression-free survival do not always reflect long-term survival benefit in melanoma immunotherapy, while conventional response criteria may not fully capture delayed or heterogeneous patterns of response [27,144]. A similar limitation was seen in ILLUMINATE-301, where adding intratumoral tilsotolimod, a TLR9 agonist, to ipilimumab did not improve objective response rate, progression-free survival, or overall survival over ipilimumab alone in anti-PD-1-refractory advanced melanoma [137].

Taken together, these failed strategies do not argue against combination therapy in melanoma: they argue against empiric combination therapy. Their consistent lesson is that future trials should align patient selection and trial design with the resistance mechanism being targeted. This provides the rationale for the mechanism-matched and adaptive approaches discussed in Section 3.9. Selected failed or disappointing immunotherapy strategies and the corresponding lessons for future resistance-matched trial design are summarized in Table 2.

### 3.9. Rethinking Future Strategies

The strategies discussed below span different levels of clinical evidence. Some approaches, including established checkpoint combinations and treatment-sequencing strategies, are supported by randomized clinical trials in melanoma, whereas many interventions aimed at reversing specific resistance mechanisms, such as immune exclusion, myeloid suppression, or metabolic dysfunction, remain investigational and are supported primarily by preclinical or early-phase clinical data. These latter approaches should therefore be viewed as candidates for resistance-matched therapeutic development rather than established standards of care.

#### 3.9.1. Mechanism-Matched Combinations

Repeated failures of broad immunotherapy intensification in melanoma have shifted attention toward mechanism-matched combinations. Large, unselected combination trials show that biologically plausible intensification may fail when the targeted pathway is not a major resistance driver in the enrolled population [133]. The encouraging early-phase findings with bempegaldesleukin plus nivolumab, followed by failure in a randomized phase III trial, also show why signals from single-arm studies require randomized confirmation [140].

Future combinations should be linked to a suspected resistance pathway, while recognizing that current clinical tools cannot reliably identify a single dominant mechanism in every patient. The clinical efficacy of combined checkpoint blockade is illustrated by RELATIVITY-047: nivolumab plus relatlimab, targeting LAG-3 in addition to PD-1, improved progression-free survival over nivolumab alone in untreated advanced melanoma [114,145]. Importantly, benefit was observed irrespective of baseline LAG-3 expression, which did not identify patients with preferential benefit from the combination and therefore is not currently useful as a treatment-selection biomarker for nivolumab plus relatlimab [114]. However, this trial was not a biomarker-selected resistance-matched study and therefore does not establish LAG-3 inhibition as a mechanism-specific salvage strategy. Immune-excluded tumors with WNT/β-catenin activation, PTEN loss, abnormal vasculature, or stromal barriers may require approaches that improve T-cell infiltration rather than another checkpoint inhibitor [43,44,146]. Tumors with antigen presentation defects or dedifferentiated programs may need strategies that restore recognition, including epigenetic modulation, interferon pathway restoration, personalized vaccines, or cellular therapy [30,32,147]. Progress will therefore depend on resistance profiling, longitudinal sampling, and selecting combinations designed to overcome the specific barrier preventing effective antitumor immunity [30,32].

#### 3.9.2. Adaptive Approaches

Future strategies may require not only better treatments but also earlier assessment and clearer decision rules to reduce time on ineffective therapy and limit unnecessary toxicity. This approach has been most clearly demonstrated in the neoadjuvant and perioperative setting, where early pathologic response can guide treatment escalation, de-escalation, or a change in plan rather than waiting for relapse [148,149]. In PRADO, the extension cohort of OpACIN, patients with macroscopic stage III nodal melanoma received two cycles of ipilimumab plus nivolumab followed by resection of an index lymph node to assess response [148]. Subsequent treatment was based on the degree of response, with de-escalation for major responders (≤10% viable tumor) and additional local or systemic treatment for patients with a lesser response. PRADO showed high pathologic response rates and excellent 24-month relapse-free survival among major responders, though grade 3 to 4 toxicity remained clinically relevant [148].

The NADINA trial extended this concept, showing superior event-free survival with neoadjuvant ipilimumab plus nivolumab followed by response-guided adjuvant therapy compared with standard surgery followed by adjuvant nivolumab [149]. Together, PRADO and NADINA show how early treatment response can be used to adjust subsequent treatment rather than applying the same approach to all patients. A similar principle may be relevant in metastatic melanoma, where durable disease control after elective ICI discontinuation in some responders suggests that treatment intensity or duration need not be uniform; however, unlike the neoadjuvant setting, there is currently no validated pathologic response-guided de-escalation strategy, and the role of radiographic or molecular markers such as ctDNA in guiding treatment reduction remains investigational [2,3].

#### 3.9.3. Sequencing Strategies

As melanoma resistance biology has become clearer, sequencing has become more than treatment ordering; it is a decision about when each therapy is most likely to work and how prior therapy may reshape the TIME. This is especially important in BRAF-mutant melanoma, where targeted therapy and checkpoint blockade are both active but differ in speed, durability, and resistance patterns [150,151,152].

BRAF and MEK inhibitors can produce high response rates and remain important when prompt tumor control is needed, including in symptomatic or rapidly progressive BRAF mutant disease [150,153]. However, the tumor and immune changes that emerge with acquired resistance may affect the efficacy of subsequent immunotherapy [31,41,42,154]. Checkpoint blockade may produce slower responses but can provide more durable disease control in selected patients [155,156].

This is supported clinically by DREAMseq, which showed superior overall survival when nivolumab plus ipilimumab preceded dabrafenib plus trametinib rather than the reverse in advanced BRAF-mutant melanoma [46]. SECOMBIT similarly supported introducing immunotherapy early rather than waiting until after progression on targeted therapy [47]. These data do not mean that immunotherapy should always come first; sequencing should balance the durability of immune control against the urgency of tumor reduction.

Sequencing also matters after immunotherapy failure. In selected patients with oligoprogression, surgery or stereotactic radiotherapy may allow ongoing systemic treatment and delay a switch in systemic therapy [1,6]. Treatment sequencing should be individualized according to disease tempo, BRAF status, response kinetics, resistance phenotype, and longitudinal immune profiling rather than following a rigid universal algorithm.

#### 3.9.4. Tumor Microenvironment and Metabolic Targeting

If melanoma resists immunotherapy because the microenvironment remains suppressive, the next step should not be adding another immune drug. A more rational strategy is to target the suppressive pathway most likely to be driving resistance, where one can be identified, and identify patients most likely to benefit [157,158]. In PD-1-refractory disease, anti-PD-1 plus lenvatinib demonstrated objective responses and durable disease control in a subset of heavily pretreated patients, although clinically significant treatment-related toxicity remained relevant [159].

Myeloid reprogramming is another option. Early-phase evidence also exists for myeloid-directed approaches. In the phase I/1b MARIO-1 study, eganelisib, a PI3K inhibitor intended to shift suppressive myeloid cells, combined with nivolumab showed modest activity in anti-PD-1-resistant melanoma, including some durable responses, suggesting that selected resistance states remain reversible [160]. These findings, however, remain investigational and require prospective validation in biomarker-defined populations. Metabolic targeting may also require patient-level selection. Circulating melanoma-associated extracellular vesicles expressing CD39 and CD73 can generate immunosuppressive adenosine, and increased EV CD73 activity has been associated with disease progression, supporting further evaluation of the adenosine pathway as a therapeutic and biomarker target [161].

Overall, TME and metabolic targeting are most useful when the relevant mechanism can be measured and linked to prospective biomarker selection rather than applied as universal additions to PD-1 blockade [162]. In practice, such selection would likely rely on clinically tractable biomarker panels, not on comprehensive multi-omic profiling for every patient, with broader multi-omic approaches remaining investigational as discussed in Section 3.7.6.

#### 3.9.5. Next-Generation Trial Designs

Repeated failures of biologically plausible strategies show that future progress will depend not only on better therapies but on better trial design. Conventional trials often group patients under broad labels such as PD-1-resistant disease, even though this label spans the biologically distinct resistance states described above [32,99,163].

Future trials should incorporate resistance biology prospectively rather than treat biomarkers as retrospective explanations. Multi-omic profiling, spatial analysis, ctDNA kinetics, immune phenotyping, and serial sampling may help define distinct resistance states and guide the selection of mechanism-specific interventions [7,141]. Adaptive and platform designs are especially attractive because they can evaluate multiple resistance-targeted strategies, stop ineffective arms early, and expand promising cohorts [142,143].

Endpoint selection also needs refinement. Radiographic response alone may miss delayed responses, dissociated responses, durable stabilization, or early biologic evidence of benefit [22,27,164]. Trial populations should also better reflect real-world melanoma patients, including those often excluded for brain metastases, autoimmune disease, organ dysfunction, concurrent immunosuppression, or poorer performance status [11]. These changes may allow future trials to determine whether a treatment is effective in the resistance state it was designed to target.

### 3.10. Implications Beyond Melanoma

#### 3.10.1. Shared Principles of ICI Resistance

Several mechanisms characterized in melanoma, including loss of antigen presentation, impaired interferon signaling, immune exclusion, alternative-checkpoint expression, suppressive myeloid and regulatory T-cell programs, and metabolic changes within the tumor microenvironment, have also been observed in other solid tumors [8,30,42,43,99,165,166].

In non-small cell lung cancer, acquired resistance has been linked to neoantigen evolution and impaired HLA class I antigen processing and presentation [167,168]. Clear-cell renal cell carcinoma similarly shows how angiogenic signaling, immune dysfunction, and suppressive microenvironmental programs contribute to the development of resistance to immunotherapy and antiangiogenic strategies [165]. These findings suggest that resistance mechanisms may be shared across cancers, even though the underlying tumor biology and clinical presentation differ.

#### 3.10.2. Limits of Generalization

Similarity in resistance mechanisms does not mean that interventions can be directly transferred across cancers, since the timing, dominance, measurability, and therapeutic relevance of these mechanisms may differ between tumor types [99,163]. Several features make melanoma an imperfect universal model, including its high mutational burden, strong immunogenicity, and marked phenotypic plasticity [7,29,31,120].

## 4. Discussion

Across the preceding sections, three claims emerge consistently. First, immunotherapy resistance in melanoma is not one biological event but a family of distinct states. Second, these states present through clinical patterns that do not map cleanly onto one another: primary resistance, acquired resistance, early progression, and relapse after discontinuation may each reflect different combinations of mechanisms [1,4], while dissociated response and brain-only progression show that resistance can be lesion-specific or anatomically restricted [5,18]. Third, trials that have failed to improve on checkpoint blockade often share a structural problem: they test broad immune intensification in biologically unselected populations, dilute mechanism-specific signals across heterogeneous patients, and rely on endpoints that may not capture the kinetics of the intervention being tested [133,138,141].

Resistance can be mapped along three axes. The first is the clinical pattern at progression: oligoprogression and dissociated response suggest lesion-level escape, whereas diffuse multisite progression suggests broader systemic resistance [1,5,6]. The second is the dominant biological barrier: a tumor that has lost B2M expression or JAK signaling requires a different rescue strategy from one in which T cells are abundant but exhausted or immune exclusion is maintained by stromal and vascular barriers [8,30,33]. The third is the intervention class. Local therapy may preserve systemic immune benefit when progression is anatomically limited, whereas systemic treatment may be informed by the suspected resistance biology whenever it is supported by clinically interpretable biomarkers [6]. Endpoint selection should also reflect the biological effect the intervention is expected to produce rather than statistical convenience alone.

This framework does not promise that every melanoma will fit cleanly into one category. Lesions within the same patient may differ, and resistance phenotypes may evolve under treatment pressure [7,16,121]. The value of this framework is therefore practical: future combinations, treatment sequences, and trials should define the resistance phenotype they intend to address [99], confirm target engagement [131,141], and select endpoints that reflect the expected treatment effect [141,144]. Mechanism-matched design connects clinical progression, resistance biology, and trial outcomes and should guide future research in post-PD-1 melanoma. Primary resistance may provide a particularly useful clinical starting point for prospective resistance-focused studies because it represents a relatively clearly defined treatment-failure phenotype, although its underlying biology remains heterogeneous. Other patterns, including acquired, oligoprogressive, mixed, and site-specific resistance, may require greater biomarker refinement to identify the mechanism driving progression and select targeted interventions.

## 5. Conclusions

Immunotherapy failure in melanoma should not be viewed as a single endpoint or a uniform biological state. The same radiographic label of progression may reflect primary resistance, acquired lesion-level escape, brain-dominant progression, antigen-presentation loss, immune exclusion, suppressive myeloid biology, metabolic restriction, or T-cell clonal failure. This heterogeneity explains why empiric immune intensification has often produced disappointing results despite strong biological rationale. Future progress will require a shift from broad salvage strategies toward resistance-matched design. Clinical patterns of failure should guide tissue, molecular, imaging, and liquid-biopsy evaluation, while interventions should be informed by the suspected biological barrier and tested with endpoints that match the expected kinetics of benefit. Melanoma remains an important model for understanding checkpoint resistance, but its lessons are best applied as a framework: define the resistance phenotype, confirm the mechanism, enrich the population, and choose endpoints that can capture durable immune control.

## Figures and Tables

**Table 1:** Biomarker and response-assessment pitfalls in melanoma immunotherapy failure.

Tool or Biomarker	What It Captures	Main Limitation	Best Use in Resistance-Matched Design
PD-L1 expression	Local adaptive immune engagement	Marked spatial and temporal heterogeneity; poor negative predictor	Enrichment tool, not a stand-alone selector
Tumor mutational burden	Neoantigen potential	Quantity of mutations does not ensure antigen quality, presentation, or immune infiltration	Combine with antigen-presentation and immune-context markers
Single-lesion tissue biopsy	Site-specific tumor and immune biology	May miss interlesional heterogeneity and evolving resistance	Most useful when taken from the progressing lesion
ctDNA	Dynamic tumor burden and clonal evolution	Low shedding in some sites, especially brain or subcutaneous disease, can cause false negatives	Early response monitoring, relapse detection, and resistance tracking
Composite molecular models	Integrated clinical, genomic, and transcriptomic predictors	Require assay standardization and prospective validation	Trial enrichment and hypothesis generation
iRECIST/immune-response criteria	Delayed response, pseudoprogression, and atypical kinetics	Does not replace clinical judgment; applies mainly to stable patients	Confirm progression before stopping therapy when clinically appropriate

**Table 2:** Selected failed or disappointing immunotherapy strategies in melanoma and lessons for resistance-matched trial design.

Strategy or Trial	Key Result	Lesson for Future Resistance-Matched Trials
ECHO-301, epacadostat plus pembrolizumab	No progression-free survival or overall survival benefit over pembrolizumab plus placebo in unresectable or metastatic melanoma [133]	A biologically plausible pathway is not enough without clear target engagement and patient enrichment
Addition of gp100 vaccine to ipilimumab	Ipilimumab improved overall survival compared with gp100 alone, but adding gp100 to ipilimumab did not improve survival over ipilimumab alone [134]	Added antigen exposure does not necessarily improve checkpoint blockade efficacy
Canvaxin plus BCG	No overall survival or disease-free survival improvement over BCG plus placebo after complete resection of stage IV melanoma [135]	Broad adjuvant immune stimulation may fail despite biologic rationale and favorable early-phase data
gp100 peptide vaccine plus high-dose IL-2	Improved centrally verified response rate and progression-free survival, but the overall survival difference was not statistically definitive [136]	Early response or disease control may not reliably translate into survival benefit
PIVOT IO-001, bempegaldesleukin plus nivolumab	Lower objective response rate, no progression-free survival or overall survival benefit, and higher grade 3 to 4 treatment-related adverse events versus nivolumab alone [138]	Undirected immune stimulation can add toxicity without improving tumor control
ILLUMINATE-301, tilsotolimod plus ipilimumab	No improvement in ORR or OS over ipilimumab alone in anti-PD-1-refractory advanced melanoma [137]	Local immune activation alone may not overcome anti-PD-1 resistance without biomarker-defined pathway dependence

## Data Availability

No new data were created or analyzed in this study. Data sharing does not apply to this article.

## Abbreviations

The following abbreviations are used in this manuscript:

AhR	Aryl hydrocarbon receptor
ARG1	Arginase-1
B2M	Beta-2 microglobulin
BCG	Bacillus Calmette–Guérin
CAF	Cancer-associated fibroblast
CNS	Central nervous system
ctDNA	Circulating tumor DNA
CTLA-4	Cytotoxic T-lymphocyte-associated protein 4
ECM	Extracellular matrix
FDA	Food and Drug Administration
FDG-PET	Fluorodeoxyglucose positron emission tomography
G-CSF	Granulocyte colony-stimulating factor
GM-CSF	Granulocyte–macrophage colony-stimulating factor
HIF-1α	Hypoxia-inducible factor 1 alpha
ICB	Immune checkpoint blockade
ICI	Immune checkpoint inhibitor
IDO	Indoleamine 2,3-dioxygenase
IDO1	Indoleamine 2,3-dioxygenase 1
IFN-γ	Interferon gamma
IL	Interleukin
iCPD	Immune-confirmed progressive disease
iRECIST	Immune Response Evaluation Criteria in Solid Tumors
iUPD	Immune-unconfirmed progressive disease
JAK	Janus kinase
LAG-3	Lymphocyte-activation gene 3
MAPK	Mitogen-activated protein kinase
MARCO	Macrophage receptor with collagenous structure
MDSC	Myeloid-derived suppressor cell
MHC	Major histocompatibility complex
MIF	Macrophage migration inhibitory factor
M-MDSC	Monocytic myeloid-derived suppressor cell
MRI	Magnetic resonance imaging
ORR	Objective response rate
OS	Overall survival
PD-1	Programmed cell death protein 1
PD-L1	Programmed death-ligand 1
PFS	Progression-free survival
PI3K	Phosphoinositide 3-kinase
PMN-MDSC	Polymorphonuclear myeloid-derived suppressor cell
RECIST	Response Evaluation Criteria in Solid Tumors
SITC	Society for Immunotherapy of Cancer
STAT	Signal transducer and activator of transcription
TAM	Tumor-associated macrophage
TCR	T-cell receptor
TDO	Tryptophan 2,3-dioxygenase
TGF-β	Transforming growth factor beta
TIL	Tumor-infiltrating lymphocyte
TIME	Tumor immune microenvironment
TLR	Toll-like receptor
TMB	Tumor mutational burden
TME	Tumor microenvironment
Treg	Regulatory T cell
VEGF	Vascular endothelial growth factor
